# Death from Stricture

**Published:** 1829-01-01

**Authors:** 


### XXIX.

### Death from Stricture.

The physician is not seldom suspected of accelerating the progress of his patient to the next world; while the surgeon is always defeating grim death, by means of various instruments, that would be difficult to enumerate. In the following case the surgeon was at least defeated.

Capt. E———, of the Royal Navy, aged upwards of 60 years, had been, for several years, severely afflicted with stricture of the urethra; and some of the most able surgeons of London, as well as of the West country, had tried to pass bougies into the bladder, without success. The difficulty of making water had, at length, so much increased, that he was obliged to wear a bottle, into which the urine distilled guttatim. In this state he came to London, in the latter part of July, 1828, and put himself under the care of a gentleman who has directed much of his attention to strictures, and other diseases of the urinary organs. This gentleman attempted several times to pass a bougie, but never could succeed. He used no violence. In the course of these attempts at introduction, some feverish symptoms came on, and the use of the bougie was deferred. The fever increased—hiccup and vomiting came on, and on Thursday, the 14th August, the Editor of this Journal was called in to the patient. Capt. E. had a sunken countenance — pulse 110, and sharp — tongue red and glazed—skin nearly natural—constant vomiting whenever any thing was taken into the stomach—incessant hiccup—stillicidium urinæ—urine fetid, and containing a considerable portion of muco-purulent matter. On examination, a hard round tumour was felt in the left hypogastrium, exceedingly tender to the touch, rising nearly to a level with the umbilicus, and not passing to the right of the linea alba. On introducing the finger into the rectum, a pretty firm tumour was felt there, and was supposed to be an enlarged prostate gland. As the total quantity of urine discharged in the course of the day, was reported by the medical gentleman in attendance, to be three pints, or more, Dr. J. could not determine the precise nature of the tumour in the left hypogastrium, but recommended leeches and fomentations to the part—emollient injections to be thrown up—and effervescing draughts with laudanum to be given by the mouth. By these means, the vomiting and hiccup were almost entirely dissipated—the tenderness of the hypogastrium diminished—and the size of the tumour somewhat reduced. This amelioration continued through Friday, the 15th, and the patient took some nourishment, which remained on the stomach. On Saturday, the 16th August, the excretion of urine stopped, and a round elastic tumour was felt that evening, as high as the umbilicus, and occupying *equally* the anterior portions of the right and left hypogastrium. The original tumour of the left side was involved in this one, and could not be felt separately. It was now evident that the bladder was greatly distended with urine. Fomentations—enemata—nitrous ether, nitre, and colchicum, with opium, re-established the discharge of urine, and on Sunday, the 17th, the original tumour in the left hypogastrium alone could be felt. But the hiccup and vomiting returned—the pulse rose to 130, feeble and irritable—there was much pus, and some blood in the urine—the face became hippocratic—and on Monday, the 19th, at 2 o'clock, P. M., Capt. E. expired.

*Dissection, twenty-four hours after death.* The examination was made by Mr. J. H. Johnson, in presence of two other medical gentlemen. On laying open the abdomen, there were some portions of the small intestines found to be injected and inflamed, but to a very trifling extent.

There were no adhesions indicative of peritoneal inflammation. The bladder was distended, and rose nearly as high as the umbilicus, exactly in the middle of the hypogastrium. The coats of the organ seemed much thickened, and the peritoneal was highly injected with blood, &c. The scrotum and integuments of the penis were livid and almost gangrenous. The bladder, prostate gland, a portion of rectum, and the penis, were removed entire from the body, and carefully examined in a strong light. The bladder remained distended with its contents, a very little of which could be pressed through the urethra guttatim. A puncture being made in the fundus of the bladder, about three pints of muco-purulent urine were discharged. The organ was then slit open to the neck. More than three fourths of its internal surface was completely and deeply ulcerated—the ulcerations, in most places, penetrating to the muscular coat, and merely leaving some insulated patches of the mucous membrane visible. The other coats were greatly thickened. The prostate gland was not enlarged, and the first stricture was found to be situated about an inch exterior to the caput gallinaginis. It was nearly impervious, and the *immediate* surrounding structure was condensed into a state approaching to cartilage. About an inch from this was situated the second stricture (counting from the neck of the bladder) so impervious, that the point of a probe could not penetrate it, and any vestige of the canal could not, in fact, be distinctly traced. From this point forward to the glans penis, there was no stricture of any importance. But parallel with these two strictures, and extending beyond them and before them, were found three large pouches or sacs, filled with pus and coagulable lymph, with openings into the urethra *anterior* to the strictures, and evidently the results of three *false passages* made at some remote periods. These pouches were lined with organized membranes, and one of them was nearly three inches in length, capable of holding an ounce of pus and lymph. The others were somewhat smaller, but the three surrounded the membranous part of the urethra, and were evidently in a state of recent and acute inflammation. The ureters were two or three times their natural size. There was nothing to account for the tumour that was felt in the left hypogastrium; and, therefore, it must have been the bladder, perhaps in its ordinary, though morbid state of distention. When the temporary retention of urine took place, the bladder was farther distended—assumed the middle position—rose to the umbilicus—and then the tumour disappeared from the left hypogastrium. The tumour felt in the rectum, and supposed to be the prostate gland, was also the bladder, whose thickened coats conveyed a feeling very different from that of a healthy and distended bladder.

The case now recorded, conveys a salutary lesson to surgeons, and ought to make them a little cautious in the use of instruments, for the cure of stricture. It is not a little curious, that Capt. E. had been under *three* of the very first surgeons in London—and sure enough there were *three false passages!* It is not meant to connect these events together in the chain of cause and effect—they were mere coincidences—but they are very curious ones. That the false passages, the infiltration of urine into these passages, the necessary inflammation that ensued, and the formation of three pouches (constantly secreting pus and coagulable lymph) around that part of the urethra, in which the strictures were situated, must have contributed, not a little, to aggravate the original strictures, and thus render the passage utterly impermeable in the end, there can be little doubt. It is difficult to account for the extensive ulceration of the bladder, and the little inconvenience which it seemed to produce, the patient complaining but little of pain in the region of the bladder.

---

